# A Causal Relationship Between Type 1 Diabetes and Risk of Osteoporosis: A Univariable and Multivariable Mendelian Randomization Study

**DOI:** 10.1155/2024/1610688

**Published:** 2024-05-08

**Authors:** Hailin Qin, Kui Yang, Hufei Wang, Wenyong Jiao

**Affiliations:** ^1^Department of Orthopedics Surgery, The First People's Hospital of Hechi, No. 124, Guiyu Street, Yizhou District, Hechi City 546300, Guangxi Zhuang Autonomous Region, China; ^2^Department of Orthopedics Surgery, The Second Affiliated Hospital of Ning Xia Medical University, No. 2 Liqun Street, Yinchuan 750000, Ningxia, China

**Keywords:** Mendelian randomization study, osteoporosis, type 1 diabetes

## Abstract

**Objective:** This Mendelian randomization (MR) analysis aims to investigate the causal relationship between type 1 diabetes (T1D) and osteoporosis (OP).

**Methods:** Single nucleotide polymorphisms (SNPs) associated with T1D were selected from the summary statistics of the genome-wide association study (GWAS) in European ancestry as instrumental variables (IVs) for univariable MR (UVMR) to explore the causal relationship between T1D and OP. Inverse variance weighting (IVW) was the primary method used to assess possible causality between T1D and OP. MR-PRESSO and MR-Egger intercepts were used to assess the horizontal pleiotropy of the IVs, and *Q* tests and the “leave-one-out” method were used to test for heterogeneity of MR results. Multivariable MR (MVMR) analysis was used to account for potential confounders such as smoking, obesity, drinking, and serum 25-hydroxyvitamin D (25OHD) concentrations.

**Result:** Inverse variance weighted estimates suggest T1D may increase risk of OP (UVMR: OR = 1.06, 95% CI: 1.02–1.10, *p* = 0.002) (MVMR: OR = 1.50, 95% CI: 1.07–1.90, *p* < 0.001).

**Conclusion:** Our findings suggest that T1D can increase the risk of OP.

## 1. Introduction

Osteoporosis (OP) is a disease of imbalanced bone metabolism characterized by a generalized decrease in bone mass, which can lead to complications such as fractures, pain, and skeletal deformities [1]. The occurrence of OP is related to a variety of factors, and previous studies have suggested a strong link between diabetes and OP, but most studies have focused on the relationship between type 2 diabetes and OP [2, 3]. In some studies, it has been shown that bone mineral density can be increased in obese patients with type 2 diabetes, but the risk of osteoporotic fracture is not reduced [4]. Therefore, some scholars believe that BMD values in patients with type 2 diabetes do not reflect their risk of fracture [5]. However, there are relatively few studies on the association between type 1 diabetes (T1D) and OP. And because of the susceptibility to residual or reverse causality, observational studies may be biased by residual confounding, whereas Mendelian randomization (MR) analyses using genetic variation as instrumental variables (IVs) to test the causal relationship between risk factors and disease can reduce some of the potential confounding and avoid reverse causality bias [6]. In this study, we aimed to assess the causal effect of T1D on the risk of OP using a two-sample and multivariate MR.

## 2. Method

We utilized summary-level data obtained from publicly available genome-wide association studies (GWAS) for each of the traits listed in Table S1. We obtained genetic IVs for T1D from a meta-analysis that included 12 cohorts of European ancestry (9266 cases and 15,574 controls) [7]. Based on previous studies, we selected smoking, alcohol consumption, obesity, and serum 25-hydroxyvitamin D (25OHD) as confounders for the multivariate MR study. IVs for smoking and drinking were obtained from a meta-analysis of risk behaviors that included the GWAS of every smoker (*n* = 518,633) and the GWAS of drinks per week (*n* = 414,343) [8]. Genetic IVs for obesity (4688 cases and 458,322 controls) were obtained from GWAS in the UK Biobank. IVs for serum 25OHD concentration were obtained from a public genome-wide association study (*n* = 417,580) [9]. GWAS summary data for OP are available from the FinnGen Consortium and include 3203 cases and 209,575 controls (https://www.finngen.fi/en). Single nucleotide polymorphisms (SNPs) that reached genome-wide significance (*p* < 5 × 10^−^^8^) were used as IVs. We then selected a reference sample of European ancestral individuals formed from 1000 genome projects to estimate allele frequencies and levels of linkage disequilibrium (LD) [10]. IVs were clumped within a genetic window of 10,000 using a strict LD threshold of *r*^2^ = 0.001 to determine that SNPs were independent. We also calculated the *F*-statistics of the SNPs to determine the strength of the instruments, with *F*-statistics > 10 [11]. There was no overlap in samples between exposure and outcome variables.

## 3. Statistical Methods

We performed two-sample MR analyses to test the potential causal relationship between T1D and OP risk. The inverse variance weighting (IVW) method was used as the primary method of analysis, with a *p* value of < 0.05 indicating a statistically significant causal relationship between T1D and OP [12]. MR-PRESSO and MR-Egger were used for the detection of horizontal pleiotropy [13, 14]. Cochran's *Q* test and MR-Egger regression in the IVW method were used to test for heterogeneity of genetic instruments in the T1D GWAS dataset, with *p* values > 0.05 indicating no statistically significant pleiotropy or heterogeneity. The effect of each IV on the risk of OP was evaluated using a leave-one-out sensitivity analysis. We further performed multivariable MR analysis with T1D, smoking, obesity, alcohol consumption, and serum 25OHD as exposure factors and OP as the outcome.

## 4. Result

**Figure 1:**
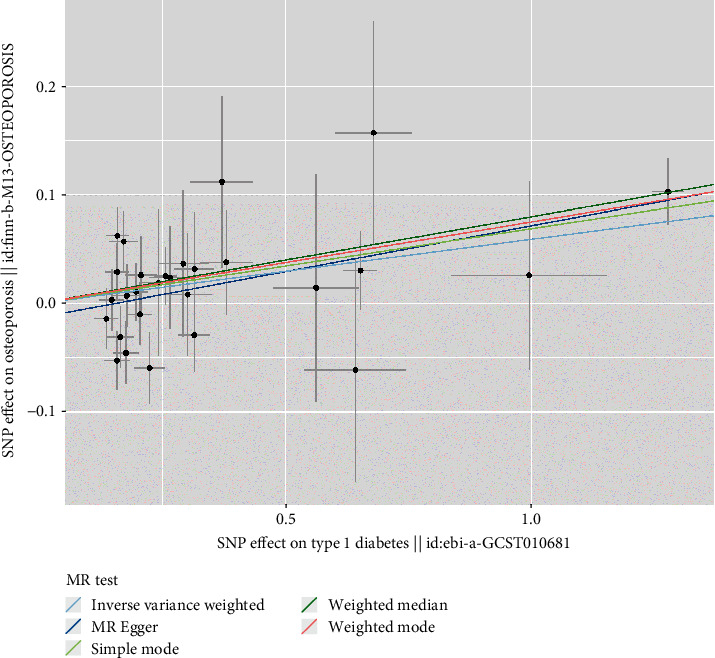
Scatter plots for the causal association between type 1 diabetes and osteoporosis.

**Figure 2:**
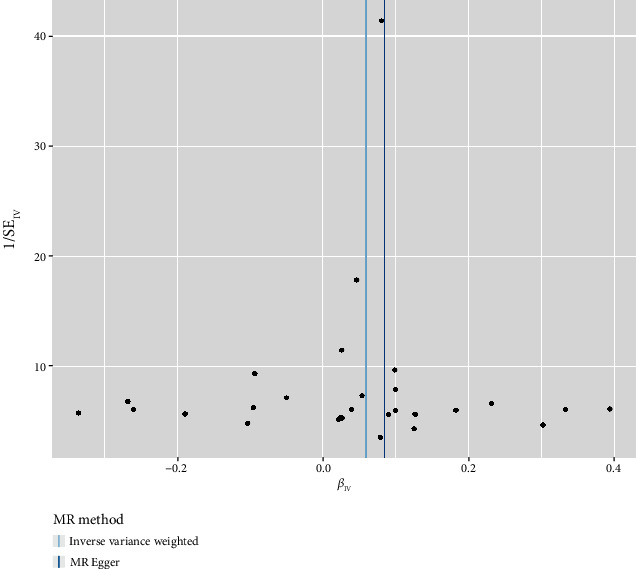
MR funnel plot of IVW and MR-Egger methods.

**Figure 3:**
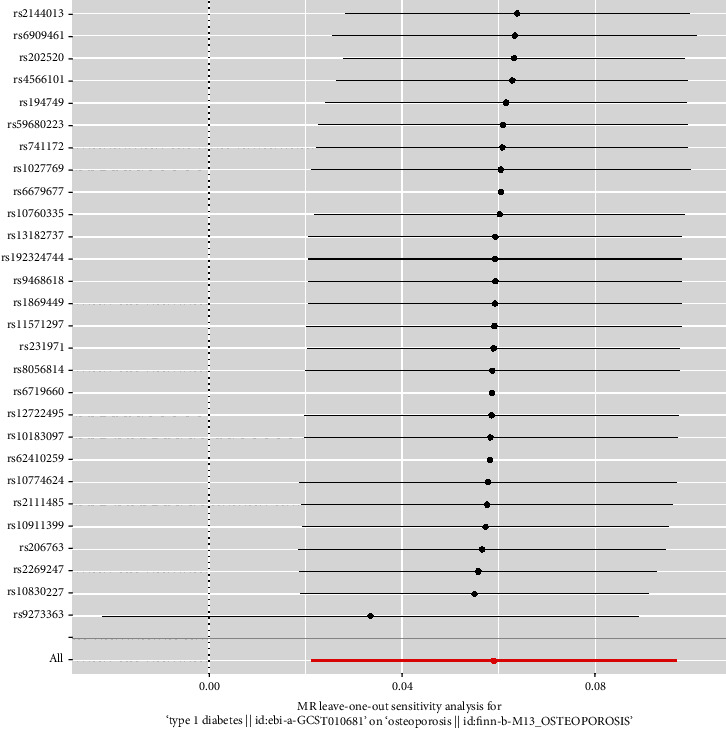
Leave-one-out plots for the causal association between type 1 diabetes and osteoporosis.

We finally identified 28 independent SNPs significantly associated with T1D as IVs (Table S2). All IVs had *F*-statistics > 10, excluding weak instrumental bias and satisfying the hypothesis that IVs are strongly associated with exposure factors. The results of IVW showed a causal relationship between T1D and increased risk of OP (OR = 1.06, 95% CI: 1.02–1.10, *p* = 0.002). MR-Egger, weighted median, and weighted mode were consistent with the IVW results. Simple mode did not show this relationship (Table 1 and Figure 1). The MR-Egger and MR-PRESSO results did not show the presence of horizontal pleiotropy (*p* > 0.05) (Table 2). Cochran's *Q* test showed no significant heterogeneity in these IVs (Table 3). The symmetrical distribution of funnel plots shows no significant heterogeneity (Figure 2). All IVs in T1D are stable and associated with OP. The leave-one-out method did not identify SNP that could significantly alter the results (Figure 3). Table S3 provides a detailed breakdown of the IVs employed in the multivariable MR study. When MVMR analysis was performed, the effect estimate of T1D on the risk associated with OP was significantly increased (MVMR: OR = 1.50, 95% CI: 1.07–1.90, *p* < 0.001) (Table 4).

## 5. Discussion

We concluded that T1D is a risk factor for OP from a genetic point of view by analyzing the MR of both samples. As the aging process progresses, the metabolic homeostasis of the skeleton decreases and the acceleration of bone loss leads to the development of OP. There are many diseases that play a role in the development of OP. T1D is characterized by insulin deficiency due to depletion of pancreatic B-cells, while type 2 diabetes is characterized by elevated blood insulin in the early stages due to insulin resistance, and therefore it has been hypothesized that insulin promotes the metabolic synthesis of bone.

Danielson et al. [15] showed that poor glycemic control may be a risk factor for reduced BMD in menopausal T1D patients with impaired bone formation and resorption conversion. However, in a recent meta-analysis, it was shown that there was no significant difference in early BMD in adult T1D patients compared to the normal population [16]. Campos Pastor et al. [17] showed that the presence of retinopathy was associated with the progression of bone loss in diabetic patients with good glycemic control. Halper-Stromberg et al. [18] found a statistically significant trend toward lower BMD in patients with T1D in a population of postmenopausal women, whereas this difference was not statistically significant in other age groups. Therefore, the degree of BMD loss in patients with T1D may be related to age and sex as well as the duration of disease presence.

Although a number of observations have shown that BMD becomes elevated in T2D and decreases in T1D, both have significantly higher fracture risk than the nonosteoporotic population [19]. In one study, the bone cortex of the femoral neck was shown to be thinner in patients with T1D than in the normal population [20]. Impaired bone microarchitecture is more pronounced in patients with microvascular disease [21]. T1D patients with poor glycemic control have lower fracture conversion and lower levels of bone resorption, suggesting that hyperglycemia may inhibit bone metabolism [22, 23].

Our study used two-sample and multivariable MR analyses to explore the causal relationship between T1D and OP. MR modeling was used to control for the influence of confounders on the estimates, thereby obtaining reliable estimates of causal effects based on observational studies. Finally, MR methods are less likely to be affected by confounders or reverse causality than traditional observational studies, and thus our results provide more compelling evidence in support of a causal relationship between T1D and OP.

There are some limitations to this study. First, despite our use of the MR-Egger method, pleiotropy of SNPs could not be completely excluded. Second, the SNPs used were from a European population, which may lead to bias. It is unclear whether these results can be directly applied to other populations, and therefore more comprehensive studies of different ethnic groups should be conducted. Third, because SNPs may also be associated with confounding factors, MR analyses based on genome-wide association analysis data may overestimate the association between genetics and exposure. In addition, further basic biological studies and randomized controlled trials are needed to validate the results of this study.

## Figures and Tables

**Table 1:** Two-sample Mendelian randomization analysis of the association of type 1 diabetes with the risk of osteoporosis.

**Exposure-outcome**	**No. of SNP**	**Methods**	**OR (95% CI)**	**p** **value**
T1D-osteoporosis	28	MR-Egger	1.08 (1.03 to 1.15)	0.006
		Weighted median	1.08 (1.03 to 1.13)	< 0.001
		Inverse variance weighted	1.06 (1.02 to 1.10)	0.002
		Simple mode	1.07 (0.97 to 1.19)	0.201
		Weighted mode	1.08 (1.03 to 1.12)	0.002

**Table 2:** Horizontal pleiotropy test.

**Exposure**	**Outcome**	**Egger intercept**	**Intercept ** **p** **value**	**MR-PRESSO global test ** **p** **value**	**Main MR results ** **p** **value**
T1D	Osteoporosis	−0.013	0.229	0.196	0.004

**Table 3:** Heterogeneity test.

**Exposure**	**Outcome**	**IVW**		**MR-Egger**	
		**Cochran's ** **Q**	**Q**-**pvalue**	**Cochran's ** **Q**	**Q**-**pvalue**
T1D	Osteoporosis	31.667	0.245	29.919	0.271

**Table 4:** The results of MVMR analysis.

**Exposure**	**SNP**	**OR**	**OR_low (95% CI)**	**OR_up (95% CI)**	**p** **value**
Type I diabetes	24	1.50	1.07	1.90	< 0.001
Smoke	120	1.15	0.89	1.49	0.285
Drinks per week	42	0.91	0.65	1.26	0.551
25OHD	69	1.07	0.91	1.26	0.423
Obesity	2	128.70	0.09	182712.30	0.190

## Data Availability

Data used in this study are all publicly available.

## Supporting Information

Supporting Information Additional supporting information can be found online in the Supporting Information section. Table S1: data sources used to identify genetic variants in this study. Table S2: detailed information on instrumental variables in univariate Mendelian randomization studies. Table S3: the detailed information of the instrumental variables used in MVMR.
