# Supplementary material for: A Causal Relationship Between Type 1 Diabetes and Risk of Osteoporosis: A Univariable and Multivariable Mendelian Randomization Study
Source: J Diabetes Res. 2024 May 8;2024:1610688. doi: 10.1155/2024/1610688 (PMC11095986; doi:10.1155/2024/1610688)
Supplement: Supporting Information — Additional supporting information can be found online in the Supporting Information section. Table S1: data sources used to identify genetic variants in this study. Table S2: detailed information on instrumental variables in univariate Mendelian randomization studies. Table S3: the detailed information of the instrumental variables used in MVMR. [file 1610688.f1.doc]

**Supplementary Material**

Table S1 Data sources used to identify genetic variants in this study

| **Phenotype** | **Data source** | **year** | **Category** | **Cohorts** | **Sample size** | **ID** |
| --- | --- | --- | --- | --- | --- | --- |
| **Type1 diabetes** | **Forgetta V et al** | **2020** | **Binary** |  | **24,840** | **GCST010681** |
| **Ever smoker** | **Karlsson Linnér R et al** | **2019** | **Binary** | **UKB; TAG Consortium** | **518,633** | **GCST007327** |
| **Drinks per week** | **Karlsson Linnér R et al** | **2019** | **Binary** | **UKB** | **414,343** | **GCST007328** |
| **25OHD** | **Revez et al** | **2020** | **Continuous** | **UKB** | **417,580** |  |
| **Obesity** | **Ben Elsworth** | **2018** | **Binary** | **UKB** | **463,010** | **ukb-b-15541** |
| **Osteoporosis** | **FinnGen** | **2021** | **Binary** | **FinnGen** | **212,778** | **finn-b-M13_OSTEOPOROSIS** |

Table S2 Detailed information on instrumental variables in univariate Mendelian randomization studies

| SNP | P-Value | EA | OA | Beta | SE | F |
| --- | --- | --- | --- | --- | --- | --- |
| rs10183097 | 1.82E-10 | C | T | 0.2053 | 0.0322 | 40.64725168 |
| rs1027769 | 3.52E-10 | T | G | -0.9962 | 0.1588 | 39.35112111 |
| rs10760335 | 2.43E-08 | G | A | 0.1357 | 0.0243 | 31.18258963 |
| rs10774624 | 1.34E-25 | A | G | -0.2556 | 0.0244 | 109.725376 |
| rs10830227 | 1.02E-11 | A | G | 0.1582 | 0.0233 | 46.09630851 |
| rs10911399 | 6.75E-09 | G | A | -0.3707 | 0.064 | 33.54673479 |
| rs11571297 | 1.11E-16 | C | T | -0.1964 | 0.0237 | 68.66751106 |
| rs12722495 | 1.27E-14 | C | T | -0.3145 | 0.0408 | 59.41361869 |
| rs13182737 | 1.49E-08 | A | G | 0.1465 | 0.0259 | 31.99195295 |
| rs1869449 | 4.55E-11 | A | G | 0.1769 | 0.0269 | 43.243032 |
| rs192324744 | 1.36E-10 | G | T | 0.562 | 0.0875 | 41.24977237 |
| rs194749 | 5.37E-09 | C | T | -0.1638 | 0.0281 | 33.97662104 |
| rs202520 | 7.97E-10 | G | A | -0.1573 | 0.0256 | 37.75222441 |
| rs206763 | 2.93E-18 | A | G | 0.6792 | 0.0779 | 76.01257945 |
| rs2111485 | 1.89E-10 | G | A | 0.1577 | 0.0248 | 40.43198433 |
| rs2144013 | 1.76E-12 | G | A | 0.2234 | 0.0317 | 49.66070085 |
| rs2269247 | 7.28E-09 | T | C | 0.1709 | 0.0295 | 33.55869969 |
| rs231971 | 1.55E-09 | G | A | 0.2411 | 0.0399 | 36.51015365 |
| rs4566101 | 6.23E-12 | C | T | 0.1755 | 0.0255 | 47.36296826 |
| rs59680223 | 5.00E-10 | T | C | 0.6421 | 0.1032 | 38.70891305 |
| rs62410259 | 1.02E-12 | A | G | -0.3796 | 0.0533 | 50.71810526 |
| rs6679677 | 3.42E-79 | A | C | 0.6527 | 0.0346 | 355.8279504 |
| rs6719660 | 2.52E-08 | G | A | 0.2918 | 0.0524 | 31.00794838 |
| rs6909461 | 3.06E-21 | C | A | -0.314 | 0.0332 | 89.44337124 |
| rs741172 | 3.11E-15 | T | C | -0.2034 | 0.0258 | 62.14805143 |
| rs8056814 | 1.99E-10 | A | G | 0.2641 | 0.0415 | 40.49539507 |
| rs9273363 | 1.00E-200 | A | C | 1.2786 | 0.0334 | 1465.350436 |
| rs9468618 | 7.53E-10 | T | C | -0.3009 | 0.0489 | 37.86096582 |

Table S3 The detailed information of the instrumental variables used in MVMR

| exposure | SNP | pval | EA | OA | beta | se | eaf |
| --- | --- | --- | --- | --- | --- | --- | --- |
| DRINKS PER WEEK | rs1076884 | 2.02E-08 | C | G | 0.01498 | 0.00267 | 0.223345 |
| DRINKS PER WEEK | rs113443718 | 2.52E-15 | A | G | -0.01907 | 0.00241 | 0.282091 |
| DRINKS PER WEEK | rs11604680 | 7.24E-17 | A | G | 0.01994 | 0.00239 | 0.684724 |
| DRINKS PER WEEK | rs11692435 | 1.89E-10 | A | G | 0.02567 | 0.00403 | 0.0855867 |
| DRINKS PER WEEK | rs11773627 | 9.75E-09 | T | C | -0.01623 | 0.00283 | 0.779674 |
| DRINKS PER WEEK | rs11940694 | 1.98E-41 | A | G | -0.03074 | 0.00228 | 0.405159 |
| DRINKS PER WEEK | rs12124523 | 1.78E-10 | T | C | -0.0229 | 0.00359 | 0.0928241 |
| DRINKS PER WEEK | rs1260326 | 6.00E-53 | T | C | -0.03492 | 0.00228 | 0.40482 |
| DRINKS PER WEEK | rs13107325 | 1.13E-24 | T | C | -0.04358 | 0.00425 | 0.065445 |
| DRINKS PER WEEK | rs13413953 | 2.86E-09 | T | G | 0.01378 | 0.00232 | 0.657237 |
| DRINKS PER WEEK | rs141769737 | 3.06E-09 | A | G | 0.02075 | 0.0035 | 0.113428 |
| DRINKS PER WEEK | rs142488468 | 1.89E-09 | C | G | -0.01736 | 0.00289 | 0.164429 |
| DRINKS PER WEEK | rs1568450 | 1.40E-10 | A | G | -0.01482 | 0.00231 | 0.359532 |
| DRINKS PER WEEK | rs17601612 | 2.70E-13 | C | G | -0.01681 | 0.0023 | 0.355297 |
| DRINKS PER WEEK | rs17802617 | 3.57E-08 | A | T | -0.0151 | 0.00274 | 0.805698 |
| DRINKS PER WEEK | rs1788030 | 4.97E-09 | T | C | -0.0131 | 0.00224 | 0.492023 |
| DRINKS PER WEEK | rs17884691 | 1.01E-09 | A | G | -0.01582 | 0.00259 | 0.220711 |
| DRINKS PER WEEK | rs2079227 | 3.26E-08 | A | G | 0.01238 | 0.00224 | 0.46466 |
| DRINKS PER WEEK | rs2274793 | 8.39E-09 | T | C | -0.01371 | 0.00238 | 0.328811 |
| DRINKS PER WEEK | rs2520099 | 1.15E-08 | T | C | -0.01438 | 0.00252 | 0.684401 |
| DRINKS PER WEEK | rs2858088 | 5.65E-10 | A | G | -0.01426 | 0.0023 | 0.387388 |
| DRINKS PER WEEK | rs303753 | 3.84E-08 | A | G | -0.01292 | 0.00235 | 0.341038 |
| DRINKS PER WEEK | rs322776 | 2.29E-08 | T | G | -0.01263 | 0.00226 | 0.417201 |
| DRINKS PER WEEK | rs34908430 | 2.32E-08 | T | C | -0.01363 | 0.00244 | 0.299322 |
| DRINKS PER WEEK | rs35572189 | 9.63E-09 | A | G | 0.01331 | 0.00232 | 0.345072 |
| DRINKS PER WEEK | rs35807116 | 1.15E-09 | T | C | 0.01388 | 0.00228 | 0.562165 |
| DRINKS PER WEEK | rs4775792 | 4.04E-08 | T | G | 0.01268 | 0.00231 | 0.383862 |
| DRINKS PER WEEK | rs4815364 | 4.76E-09 | A | G | 0.01335 | 0.00228 | 0.62838 |
| DRINKS PER WEEK | rs485425 | 4.82E-12 | C | G | -0.01548 | 0.00224 | 0.464721 |
| DRINKS PER WEEK | rs4916723 | 1.11E-11 | A | C | 0.01535 | 0.00226 | 0.596212 |
| DRINKS PER WEEK | rs528301 | 8.81E-20 | A | G | 0.02039 | 0.00224 | 0.605266 |
| DRINKS PER WEEK | rs62055546 | 6.62E-25 | A | C | 0.02762 | 0.00268 | 0.797582 |
| DRINKS PER WEEK | rs62305782 | 1.03E-20 | T | C | 0.03248 | 0.00348 | 0.114383 |
| DRINKS PER WEEK | rs62466318 | 7.50E-10 | T | C | 0.01705 | 0.00277 | 0.193702 |
| DRINKS PER WEEK | rs7132908 | 3.94E-08 | A | G | -0.01258 | 0.00229 | 0.367431 |
| DRINKS PER WEEK | rs7206303 | 1.76E-08 | A | G | 0.01476 | 0.00262 | 0.23899 |
| DRINKS PER WEEK | rs7620901 | 7.53E-09 | T | C | -0.01306 | 0.00226 | 0.412735 |
| DRINKS PER WEEK | rs7630012 | 6.46E-09 | A | G | 0.01306 | 0.00225 | 0.539298 |
| DRINKS PER WEEK | rs7688435 | 1.75E-08 | T | C | -0.0182 | 0.00323 | 0.156575 |
| DRINKS PER WEEK | rs77294902 | 4.66E-09 | A | G | -0.01576 | 0.00269 | 0.213366 |
| DRINKS PER WEEK | rs780569 | 3.92E-10 | A | T | -0.01533 | 0.00245 | 0.726132 |
| DRINKS PER WEEK | rs78119163 | 4.02E-08 | A | G | 0.01905 | 0.00347 | 0.898029 |
| DRINKS PER WEEK | rs838145 | 1.72E-11 | A | G | -0.01507 | 0.00224 | 0.584278 |
| DRINKS PER WEEK | rs9349379 | 4.42E-09 | A | G | -0.01332 | 0.00227 | 0.601063 |
| DRINKS PER WEEK | rs974883 | 8.41E-09 | T | C | -0.0144 | 0.0025 | 0.247167 |
| DRINKS PER WEEK | rs9822731 | 3.27E-15 | T | C | -0.02104 | 0.00267 | 0.781783 |
| Ever smoke | rs1004787 | 4.01E-16 | A | G | 0.01766 | 0.00217 | 0.580674 |
| Ever smoke | rs10252114 | 3.14E-08 | T | C | -0.01234 | 0.00223 | 0.601894 |
| Ever smoke | rs10461104 | 1.53E-08 | A | G | 0.01273 | 0.00225 | 0.622113 |
| Ever smoke | rs10751226 | 2.72E-08 | T | C | -0.01473 | 0.00265 | 0.816523 |
| Ever smoke | rs10905461 | 2.04E-10 | T | C | 0.01456 | 0.00229 | 0.281691 |
| Ever smoke | rs10914684 | 1.31E-12 | A | G | -0.01518 | 0.00214 | 0.338112 |
| Ever smoke | rs10952199 | 5.78E-10 | T | C | -0.01357 | 0.00219 | 0.417847 |
| Ever smoke | rs10984475 | 1.36E-08 | A | C | -0.01238 | 0.00218 | 0.428226 |
| Ever smoke | rs1109480 | 1.01E-09 | A | G | -0.01362 | 0.00223 | 0.381706 |
| Ever smoke | rs11103667 | 6.79E-10 | T | C | 0.01697 | 0.00275 | 0.199677 |
| Ever smoke | rs11166986 | 4.47E-09 | A | G | 0.01179 | 0.00201 | 0.496889 |
| Ever smoke | rs11210892 | 3.45E-13 | A | G | -0.01557 | 0.00214 | 0.647428 |
| Ever smoke | rs11738110 | 6.99E-14 | T | G | 0.01565 | 0.00209 | 0.354897 |
| Ever smoke | rs1174864 | 1.02E-08 | A | G | 0.01157 | 0.00202 | 0.54036 |
| Ever smoke | rs11771982 | 4.44E-09 | T | C | -0.01631 | 0.00278 | 0.13648 |
| Ever smoke | rs11783093 | 7.99E-22 | T | C | -0.02592 | 0.0027 | 0.151755 |
| Ever smoke | rs11872397 | 1.74E-09 | A | G | -0.01487 | 0.00247 | 0.252325 |
| Ever smoke | rs11876432 | 6.73E-09 | C | G | 0.01345 | 0.00232 | 0.784124 |
| Ever smoke | rs12030183 | 4.07E-12 | T | C | 0.01491 | 0.00215 | 0.682907 |
| Ever smoke | rs12042107 | 5.39E-11 | T | C | 0.0143 | 0.00218 | 0.47279 |
| Ever smoke | rs12211126 | 1.11E-09 | T | C | -0.01243 | 0.00204 | 0.629366 |
| Ever smoke | rs1221973 | 2.59E-11 | T | C | -0.01427 | 0.00214 | 0.684232 |
| Ever smoke | rs12456711 | 4.07E-08 | A | G | -0.0135 | 0.00246 | 0.742793 |
| Ever smoke | rs1246265 | 3.52E-11 | T | C | -0.01563 | 0.00236 | 0.291792 |
| Ever smoke | rs12476173 | 3.65E-08 | A | G | 0.01151 | 0.00209 | 0.625916 |
| Ever smoke | rs12517438 | 4.62E-08 | T | G | -0.01104 | 0.00202 | 0.506868 |
| Ever smoke | rs12551403 | 1.97E-09 | T | C | -0.01878 | 0.00313 | 0.101786 |
| Ever smoke | rs1271272 | 2.77E-08 | A | G | -0.012 | 0.00216 | 0.323114 |
| Ever smoke | rs12770479 | 2.03E-08 | A | G | -0.0115 | 0.00205 | 0.356775 |
| Ever smoke | rs12787182 | 1.87E-09 | A | G | 0.01328 | 0.00221 | 0.721512 |
| Ever smoke | rs12895462 | 4.80E-08 | T | C | 0.01381 | 0.00253 | 0.818771 |
| Ever smoke | rs12930834 | 6.12E-09 | A | C | 0.01372 | 0.00236 | 0.780828 |
| Ever smoke | rs13187930 | 6.10E-10 | T | C | 0.01417 | 0.00229 | 0.239082 |
| Ever smoke | rs13237637 | 5.84E-14 | C | G | -0.0163 | 0.00217 | 0.47433 |
| Ever smoke | rs13258512 | 2.39E-13 | A | G | 0.01487 | 0.00203 | 0.578904 |
| Ever smoke | rs13357015 | 1.54E-09 | A | G | 0.01359 | 0.00225 | 0.634617 |
| Ever smoke | rs13396935 | 3.60E-13 | A | G | -0.01912 | 0.00263 | 0.173899 |
| Ever smoke | rs1368550 | 1.05E-21 | T | C | 0.01924 | 0.00201 | 0.52627 |
| Ever smoke | rs1373129 | 2.82E-10 | A | C | -0.01268 | 0.00201 | 0.51389 |
| Ever smoke | rs1435741 | 7.07E-15 | A | G | 0.0158 | 0.00203 | 0.424869 |
| Ever smoke | rs143909875 | 1.54E-08 | T | C | -0.02014 | 0.00356 | 0.896689 |
| Ever smoke | rs1445585 | 3.49E-09 | C | G | -0.01394 | 0.00236 | 0.220003 |
| Ever smoke | rs1469908 | 5.50E-12 | T | C | -0.01406 | 0.00204 | 0.557191 |
| Ever smoke | rs1492546 | 8.54E-11 | C | G | -0.01415 | 0.00218 | 0.432984 |
| Ever smoke | rs1527345 | 2.24E-08 | T | C | -0.01275 | 0.00228 | 0.76891 |
| Ever smoke | rs16951001 | 3.29E-09 | T | G | 0.01207 | 0.00204 | 0.401509 |
| Ever smoke | rs16972552 | 2.14E-08 | C | G | -0.01753 | 0.00313 | 0.879997 |
| Ever smoke | rs17151637 | 2.51E-11 | T | C | -0.01488 | 0.00223 | 0.264506 |
| Ever smoke | rs17348216 | 4.02E-09 | T | C | -0.01565 | 0.00266 | 0.17761 |
| Ever smoke | rs176644 | 2.44E-09 | T | G | -0.01217 | 0.00204 | 0.418586 |
| Ever smoke | rs17733784 | 1.75E-10 | T | C | 0.01321 | 0.00207 | 0.622313 |
| Ever smoke | rs1863161 | 2.23E-09 | A | G | 0.01208 | 0.00202 | 0.5413 |
| Ever smoke | rs1891196 | 9.76E-10 | A | G | -0.01241 | 0.00203 | 0.557345 |
| Ever smoke | rs1899896 | 9.17E-10 | T | C | 0.01341 | 0.00219 | 0.28551 |
| Ever smoke | rs1909590 | 3.71E-16 | A | T | -0.02485 | 0.00305 | 0.13862 |
| Ever smoke | rs1919621 | 3.65E-12 | A | G | 0.01397 | 0.00201 | 0.525377 |
| Ever smoke | rs1931386 | 6.25E-10 | C | G | 0.01243 | 0.00201 | 0.495719 |
| Ever smoke | rs2185913 | 1.46E-08 | T | C | 0.01241 | 0.00219 | 0.723868 |
| Ever smoke | rs2202237 | 5.30E-11 | T | C | -0.01319 | 0.00201 | 0.560964 |
| Ever smoke | rs2236941 | 8.15E-09 | T | C | 0.01228 | 0.00213 | 0.349969 |
| Ever smoke | rs2240294 | 3.23E-10 | A | T | -0.0127 | 0.00202 | 0.454989 |
| Ever smoke | rs2291256 | 2.71E-09 | T | C | 0.02076 | 0.00349 | 0.0780413 |
| Ever smoke | rs2310752 | 2.48E-13 | A | G | -0.01486 | 0.00203 | 0.432199 |
| Ever smoke | rs2340403 | 1.69E-11 | T | C | -0.014 | 0.00208 | 0.600262 |
| Ever smoke | rs2358443 | 3.17E-08 | T | C | -0.01123 | 0.00203 | 0.442701 |
| Ever smoke | rs26251 | 1.10E-10 | T | G | 0.01342 | 0.00208 | 0.632492 |
| Ever smoke | rs2652434 | 2.52E-08 | T | C | -0.0112 | 0.00201 | 0.477456 |
| Ever smoke | rs2783130 | 1.46E-08 | A | G | 0.01139 | 0.00201 | 0.529612 |
| Ever smoke | rs28459916 | 3.53E-08 | A | C | -0.01731 | 0.00314 | 0.13514 |
| Ever smoke | rs2866724 | 2.73E-09 | A | G | -0.0135 | 0.00227 | 0.721743 |
| Ever smoke | rs2939756 | 2.85E-09 | A | G | -0.01194 | 0.00201 | 0.473884 |
| Ever smoke | rs301805 | 3.75E-08 | T | G | -0.01117 | 0.00203 | 0.441069 |
| Ever smoke | rs3026629 | 3.36E-08 | A | G | -0.01165 | 0.00211 | 0.343733 |
| Ever smoke | rs3099769 | 1.93E-12 | A | G | 0.01436 | 0.00204 | 0.364629 |
| Ever smoke | rs34495106 | 1.90E-20 | A | G | 0.02076 | 0.00224 | 0.377333 |
| Ever smoke | rs3783177 | 1.68E-09 | T | G | 0.0141 | 0.00234 | 0.762411 |
| Ever smoke | rs3800227 | 5.00E-09 | A | G | -0.01339 | 0.00229 | 0.299292 |
| Ever smoke | rs3818987 | 2.73E-10 | T | C | -0.0137 | 0.00217 | 0.476394 |
| Ever smoke | rs3851186 | 6.90E-09 | T | C | -0.01541 | 0.00266 | 0.225547 |
| Ever smoke | rs3980078 | 3.33E-08 | A | G | -0.01563 | 0.00283 | 0.198229 |
| Ever smoke | rs42417 | 2.67E-11 | T | C | 0.01566 | 0.00235 | 0.681337 |
| Ever smoke | rs4312833 | 3.77E-08 | A | T | -0.01942 | 0.00353 | 0.100231 |
| Ever smoke | rs4479577 | 9.88E-09 | T | C | 0.01244 | 0.00217 | 0.494734 |
| Ever smoke | rs4543592 | 2.48E-10 | T | C | -0.01272 | 0.00201 | 0.531814 |
| Ever smoke | rs4664442 | 2.02E-15 | A | G | 0.01596 | 0.00201 | 0.423622 |
| Ever smoke | rs4680392 | 3.53E-09 | T | C | -0.01364 | 0.00231 | 0.300308 |
| Ever smoke | rs4737525 | 3.92E-11 | A | G | -0.01328 | 0.00201 | 0.522652 |
| Ever smoke | rs4748779 | 1.02E-09 | T | C | -0.01374 | 0.00225 | 0.727933 |
| Ever smoke | rs4790874 | 2.81E-10 | T | C | 0.01369 | 0.00217 | 0.53611 |
| Ever smoke | rs479971 | 6.53E-10 | A | G | 0.01501 | 0.00243 | 0.302171 |
| Ever smoke | rs4814884 | 6.48E-10 | T | C | 0.01248 | 0.00202 | 0.519433 |
| Ever smoke | rs4984916 | 6.48E-13 | A | G | -0.0174 | 0.00242 | 0.255112 |
| Ever smoke | rs55921136 | 8.64E-09 | T | C | 0.01554 | 0.0027 | 0.804558 |
| Ever smoke | rs568599 | 3.79E-10 | C | G | 0.01265 | 0.00202 | 0.52316 |
| Ever smoke | rs58695218 | 4.04E-08 | A | G | 0.01515 | 0.00276 | 0.795611 |
| Ever smoke | rs597808 | 1.95E-11 | A | G | 0.01456 | 0.00217 | 0.453265 |
| Ever smoke | rs6119897 | 2.48E-12 | A | G | 0.01639 | 0.00234 | 0.252341 |
| Ever smoke | rs6265 | 3.75E-18 | T | C | -0.02215 | 0.00255 | 0.202864 |
| Ever smoke | rs6598539 | 4.33E-09 | T | C | -0.01274 | 0.00217 | 0.475962 |
| Ever smoke | rs6676022 | 2.80E-11 | T | C | -0.0221 | 0.00332 | 0.117447 |
| Ever smoke | rs6720647 | 6.63E-09 | A | G | -0.01363 | 0.00235 | 0.825685 |
| Ever smoke | rs6852117 | 6.31E-12 | C | G | 0.01402 | 0.00204 | 0.588251 |
| Ever smoke | rs6892220 | 3.05E-09 | A | G | 0.01334 | 0.00225 | 0.731321 |
| Ever smoke | rs6937734 | 4.36E-23 | T | C | -0.02553 | 0.00258 | 0.785833 |
| Ever smoke | rs6965740 | 5.73E-12 | T | G | -0.01391 | 0.00202 | 0.456391 |
| Ever smoke | rs6971782 | 3.28E-08 | T | C | -0.01188 | 0.00215 | 0.30214 |
| Ever smoke | rs7092200 | 4.38E-22 | T | C | -0.01971 | 0.00204 | 0.582784 |
| Ever smoke | rs7130826 | 1.25E-08 | T | G | 0.01281 | 0.00225 | 0.730397 |
| Ever smoke | rs7160389 | 3.41E-09 | T | C | -0.01188 | 0.00201 | 0.515553 |
| Ever smoke | rs72678864 | 3.26E-10 | A | G | -0.01804 | 0.00287 | 0.144718 |
| Ever smoke | rs7295765 | 3.80E-08 | A | C | -0.01485 | 0.0027 | 0.817585 |
| Ever smoke | rs745570 | 1.33E-08 | A | G | 0.01142 | 0.00201 | 0.489421 |
| Ever smoke | rs7553158 | 5.73E-12 | A | G | -0.01391 | 0.00202 | 0.548121 |
| Ever smoke | rs7668995 | 1.43E-13 | A | T | -0.01634 | 0.00221 | 0.276532 |
| Ever smoke | rs7679162 | 3.12E-10 | T | G | 0.01441 | 0.00229 | 0.652294 |
| Ever smoke | rs772921 | 3.83E-09 | T | C | -0.01249 | 0.00212 | 0.316123 |
| Ever smoke | rs7754741 | 1.33E-11 | T | C | -0.01529 | 0.00226 | 0.727826 |
| Ever smoke | rs77878475 | 2.74E-11 | A | T | -0.02584 | 0.00388 | 0.0661688 |
| Ever smoke | rs78175438 | 7.85E-09 | T | C | -0.0187 | 0.00324 | 0.893363 |
| Ever smoke | rs7870475 | 2.73E-10 | T | C | -0.01269 | 0.00201 | 0.510225 |
| Ever smoke | rs7901883 | 4.43E-08 | A | G | -0.01308 | 0.00239 | 0.245735 |
| Ever smoke | rs7921378 | 2.87E-18 | C | G | -0.01752 | 0.00201 | 0.463274 |
| Ever smoke | rs7938812 | 7.92E-48 | T | G | -0.02993 | 0.00206 | 0.575531 |
| Ever smoke | rs883403 | 3.41E-10 | T | C | 0.01733 | 0.00276 | 0.803234 |
| Ever smoke | rs905871 | 2.98E-08 | A | G | 0.01175 | 0.00212 | 0.683954 |
| Ever smoke | rs9388686 | 4.18E-08 | A | C | -0.01442 | 0.00263 | 0.184509 |
| Ever smoke | rs9571576 | 1.19E-08 | T | C | -0.01146 | 0.00201 | 0.504666 |
| Ever smoke | rs961414 | 5.94E-28 | T | G | -0.02203 | 0.00201 | 0.52527 |
| Ever smoke | rs962472 | 7.87E-09 | T | C | -0.0116 | 0.00201 | 0.507869 |
| Ever smoke | rs969650 | 2.26E-08 | T | C | 0.01174 | 0.0021 | 0.610887 |
| Ever smoke | rs9879903 | 6.62E-14 | A | G | 0.01544 | 0.00206 | 0.620912 |
| Obesity | rs11642015 | 6.77E-19 | T | C | 0.00188328 | 0.000212114 | 0.403654 |
| Obesity | rs7231987 | 2.17E-10 | T | G | 0.00149534 | 0.000235518 | 0.267232 |
| Type I diabetes | rs10183097 | 1.82E-10 | C | T | 0.2053 | 0.0322 | 0.1362 |
| Type I diabetes | rs10760335 | 2.35E-08 | G | A | 0.1357 | 0.0243 | 0.3205 |
| Type I diabetes | rs10774624 | 1.12E-25 | A | G | -0.2556 | 0.0244 | 0.5037 |
| Type I diabetes | rs10830227 | 1.12E-11 | A | G | 0.1582 | 0.0233 | 0.5741 |
| Type I diabetes | rs10865468 | 4.55E-09 | C | G | -0.1624 | 0.0277 | 0.2527 |
| Type I diabetes | rs1131017 | 4.63E-25 | G | C | -0.2461 | 0.0238 | 0.5803 |
| Type I diabetes | rs11571297 | 1.16E-16 | C | T | -0.1964 | 0.0237 | 0.4844 |
| Type I diabetes | rs12722495 | 1.27E-14 | C | T | -0.3145 | 0.0408 | 0.1122 |
| Type I diabetes | rs13182737 | 1.55E-08 | A | G | 0.1465 | 0.0259 | 0.2553 |
| Type I diabetes | rs17125653 | 4.68E-09 | A | T | 0.2355 | 0.0402 | 0.077 |
| Type I diabetes | rs1869449 | 4.83E-11 | A | G | 0.1769 | 0.0269 | 0.2967 |
| Type I diabetes | rs194749 | 5.57E-09 | C | T | -0.1638 | 0.0281 | 0.2455 |
| Type I diabetes | rs202520 | 8.02E-10 | G | A | -0.1573 | 0.0256 | 0.7222 |
| Type I diabetes | rs2111485 | 2.03E-10 | G | A | 0.1577 | 0.0248 | 0.6033 |
| Type I diabetes | rs2144013 | 1.82E-12 | G | A | 0.2234 | 0.0317 | 0.2093 |
| Type I diabetes | rs2269247 | 6.90E-09 | T | C | 0.1709 | 0.0295 | 0.1804 |
| Type I diabetes | rs231971 | 1.52E-09 | G | A | 0.2411 | 0.0399 | 0.1026 |
| Type I diabetes | rs4566101 | 5.89E-12 | C | T | 0.1755 | 0.0255 | 0.2697 |
| Type I diabetes | rs55996894 | 3.27E-08 | C | G | -0.1785 | 0.0323 | 0.204 |
| Type I diabetes | rs62410259 | 1.06E-12 | A | G | -0.3796 | 0.0533 | 0.077 |
| Type I diabetes | rs6679677 | 2.25E-79 | A | C | 0.6527 | 0.0346 | 0.0993 |
| Type I diabetes | rs689 | 3.98E-87 | T | A | 0.7004 | 0.0354 | 0.7109 |
| Type I diabetes | rs6909461 | 3.14E-21 | C | A | -0.314 | 0.0332 | 0.2529 |
| Type I diabetes | rs741172 | 3.18E-15 | T | C | -0.2034 | 0.0258 | 0.3205 |
| Type I diabetes | rs8056814 | 1.97E-10 | A | G | 0.2641 | 0.0415 | 0.0793 |
| Type I diabetes | rs9273363 | 1.00E-200 | A | C | 1.2786 | 0.0334 | 0.3317 |
| 25 hydroxyvitamin D | rs1047891 | 2.58E-09 | C | A | 0.0125534 | 0.00210766 | 0.684245 |
| 25 hydroxyvitamin D | rs10822145 | 1.60E-10 | C | T | 0.012578 | 0.00196683 | 0.524576 |
| 25 hydroxyvitamin D | rs10859995 | 1.11E-89 | T | C | 0.0399464 | 0.00198939 | 0.41738 |
| 25 hydroxyvitamin D | rs10908469 | 6.67E-13 | A | C | -0.0158902 | 0.00221124 | 0.730274 |
| 25 hydroxyvitamin D | rs11076175 | 9.46E-21 | A | G | -0.023978 | 0.00256672 | 0.821625 |
| 25 hydroxyvitamin D | rs11122455 | 3.42E-10 | C | G | -0.0126509 | 0.00201492 | 0.385944 |
| 25 hydroxyvitamin D | rs11182428 | 3.17E-10 | T | C | 0.0123489 | 0.00196317 | 0.479988 |
| 25 hydroxyvitamin D | rs1149597 | 1.78E-16 | C | T | -0.0215713 | 0.0026191 | 0.831307 |
| 25 hydroxyvitamin D | rs11542462 | 5.79E-16 | G | A | 0.0232523 | 0.00287287 | 0.865652 |
| 25 hydroxyvitamin D | rs11606 | 2.77E-08 | C | G | -0.0112201 | 0.00201965 | 0.574822 |
| 25 hydroxyvitamin D | rs11751024 | 3.76E-10 | C | A | 0.0125611 | 0.00200538 | 0.602667 |
| 25 hydroxyvitamin D | rs11939173 | 1.00E-200 | G | A | -0.100204 | 0.00197091 | 0.471822 |
| 25 hydroxyvitamin D | rs12056768 | 3.27E-28 | T | G | 0.021949 | 0.00199283 | 0.417109 |
| 25 hydroxyvitamin D | rs12317268 | 5.26E-14 | A | G | 0.0206326 | 0.00274181 | 0.849004 |
| 25 hydroxyvitamin D | rs12498888 | 1.32E-11 | C | A | -0.0146644 | 0.00216731 | 0.704889 |
| 25 hydroxyvitamin D | rs1260326 | 3.95E-29 | T | C | -0.0224633 | 0.00200516 | 0.393449 |
| 25 hydroxyvitamin D | rs12949853 | 1.23E-08 | G | A | -0.0144344 | 0.00253426 | 0.193274 |
| 25 hydroxyvitamin D | rs13011615 | 4.04E-09 | A | T | -0.0169587 | 0.00288288 | 0.864938 |
| 25 hydroxyvitamin D | rs13284054 | 1.82E-09 | T | C | -0.0185546 | 0.00308554 | 0.882245 |
| 25 hydroxyvitamin D | rs142158911 | 7.17E-18 | G | A | -0.0266711 | 0.00309689 | 0.885371 |
| 25 hydroxyvitamin D | rs1532085 | 3.62E-38 | A | G | -0.0260168 | 0.0020142 | 0.38512 |
| 25 hydroxyvitamin D | rs17144574 | 6.73E-11 | T | C | 0.01526 | 0.00233815 | 0.766294 |
| 25 hydroxyvitamin D | rs1792283 | 1.00E-200 | T | C | 0.097137 | 0.00253041 | 0.815529 |
| 25 hydroxyvitamin D | rs1792556 | 2.72E-15 | T | G | -0.0159064 | 0.00201268 | 0.600407 |
| 25 hydroxyvitamin D | rs1800588 | 1.50E-43 | C | T | 0.0329963 | 0.00238443 | 0.784792 |
| 25 hydroxyvitamin D | rs1858889 | 1.38E-08 | A | C | -0.0111372 | 0.00196228 | 0.498467 |
| 25 hydroxyvitamin D | rs1966478 | 6.92E-09 | T | C | 0.0123541 | 0.00213261 | 0.307039 |
| 25 hydroxyvitamin D | rs2012736 | 1.10E-40 | C | A | 0.0481921 | 0.00360843 | 0.919152 |
| 25 hydroxyvitamin D | rs2037511 | 1.07E-10 | G | A | -0.0170377 | 0.00263857 | 0.834022 |
| 25 hydroxyvitamin D | rs2060793 | 1.00E-200 | A | G | 0.0912536 | 0.00199564 | 0.404543 |
| 25 hydroxyvitamin D | rs212100 | 7.84E-136 | T | C | 0.065701 | 0.00264866 | 0.164003 |
| 25 hydroxyvitamin D | rs2123930 | 1.30E-10 | G | A | 0.014114 | 0.00219611 | 0.720639 |
| 25 hydroxyvitamin D | rs2131925 | 2.43E-27 | G | T | 0.0222318 | 0.00205242 | 0.356411 |
| 25 hydroxyvitamin D | rs222054 | 1.00E-200 | C | G | -0.0705193 | 0.00216148 | 0.698045 |
| 25 hydroxyvitamin D | rs2229742 | 5.90E-15 | G | C | 0.0251367 | 0.00322012 | 0.896533 |
| 25 hydroxyvitamin D | rs2246832 | 1.87E-18 | A | T | -0.017206 | 0.00196305 | 0.499443 |
| 25 hydroxyvitamin D | rs2297991 | 3.51E-08 | T | C | -0.0120361 | 0.00218295 | 0.279471 |
| 25 hydroxyvitamin D | rs2346264 | 8.86E-10 | A | C | 0.0147004 | 0.00239864 | 0.217301 |
| 25 hydroxyvitamin D | rs2608984 | 2.79E-15 | A | T | 0.0208623 | 0.00264074 | 0.834761 |
| 25 hydroxyvitamin D | rs2642439 | 6.18E-12 | A | G | 0.0145212 | 0.00211201 | 0.31497 |
| 25 hydroxyvitamin D | rs2659007 | 9.75E-09 | G | A | -0.0114099 | 0.00198953 | 0.549026 |
| 25 hydroxyvitamin D | rs2756119 | 2.35E-09 | G | A | -0.0121881 | 0.0020411 | 0.616734 |
| 25 hydroxyvitamin D | rs2847500 | 1.22E-13 | G | A | 0.0221038 | 0.00298092 | 0.876495 |
| 25 hydroxyvitamin D | rs2952289 | 3.22E-13 | C | T | -0.0178756 | 0.00245386 | 0.201966 |
| 25 hydroxyvitamin D | rs34177108 | 3.96E-08 | C | A | 0.0122879 | 0.00223709 | 0.732498 |
| 25 hydroxyvitamin D | rs34186890 | 2.25E-11 | A | G | 0.0149926 | 0.00224148 | 0.74029 |
| 25 hydroxyvitamin D | rs34284484 | 1.37E-08 | T | G | 0.0123129 | 0.002169 | 0.714404 |
| 25 hydroxyvitamin D | rs34726834 | 4.49E-11 | C | T | -0.0149657 | 0.00227206 | 0.747975 |
| 25 hydroxyvitamin D | rs35408430 | 5.82E-25 | C | T | 0.0213322 | 0.00206739 | 0.657829 |
| 25 hydroxyvitamin D | rs3814995 | 2.33E-09 | C | T | 0.0126428 | 0.00211677 | 0.68844 |
| 25 hydroxyvitamin D | rs3890624 | 3.19E-08 | A | G | -0.0112736 | 0.00203846 | 0.604899 |
| 25 hydroxyvitamin D | rs4121823 | 1.33E-11 | T | A | 0.0185043 | 0.00273512 | 0.154674 |
| 25 hydroxyvitamin D | rs41301394 | 7.11E-09 | C | T | -0.0126168 | 0.00217969 | 0.718135 |
| 25 hydroxyvitamin D | rs429358 | 1.76E-17 | T | C | 0.0231982 | 0.00272644 | 0.84751 |
| 25 hydroxyvitamin D | rs4364259 | 2.23E-11 | G | A | -0.0165067 | 0.0024674 | 0.797841 |
| 25 hydroxyvitamin D | rs4418728 | 7.54E-10 | G | T | -0.0121203 | 0.00196937 | 0.54832 |
| 25 hydroxyvitamin D | rs4616820 | 5.32E-10 | C | T | 0.0123353 | 0.00198653 | 0.535048 |
| 25 hydroxyvitamin D | rs512083 | 6.56E-10 | T | C | -0.0122018 | 0.00197554 | 0.539183 |
| 25 hydroxyvitamin D | rs57459725 | 1.07E-09 | C | G | 0.0176615 | 0.00289594 | 0.867087 |
| 25 hydroxyvitamin D | rs58387006 | 6.33E-09 | A | C | 0.0137174 | 0.0023619 | 0.778152 |
| 25 hydroxyvitamin D | rs6003465 | 1.19E-08 | T | C | 0.0119188 | 0.0020906 | 0.668017 |
| 25 hydroxyvitamin D | rs62012766 | 1.61E-11 | T | C | 0.0181582 | 0.00269511 | 0.842886 |
| 25 hydroxyvitamin D | rs62115743 | 3.21E-14 | C | T | -0.0274464 | 0.00361625 | 0.918487 |
| 25 hydroxyvitamin D | rs6438900 | 3.44E-09 | C | G | -0.0133459 | 0.00225847 | 0.742009 |
| 25 hydroxyvitamin D | rs6671730 | 2.25E-14 | G | A | 0.015115 | 0.00197958 | 0.565711 |
| 25 hydroxyvitamin D | rs6723486 | 4.24E-08 | C | T | 0.0111173 | 0.00202854 | 0.389437 |
| 25 hydroxyvitamin D | rs6724965 | 1.76E-10 | A | G | 0.0165826 | 0.00259881 | 0.828539 |
| 25 hydroxyvitamin D | rs6834488 | 1.96E-13 | C | T | 0.0146317 | 0.00199028 | 0.576288 |
| 25 hydroxyvitamin D | rs73413596 | 2.89E-10 | T | C | -0.0237483 | 0.00376674 | 0.926128 |
| 25 hydroxyvitamin D | rs7528419 | 1.34E-17 | A | G | -0.0200747 | 0.00235052 | 0.775305 |
| 25 hydroxyvitamin D | rs7569755 | 1.21E-09 | G | A | -0.0132378 | 0.00217782 | 0.709372 |
| 25 hydroxyvitamin D | rs77924615 | 8.09E-09 | G | A | 0.0144881 | 0.00251244 | 0.806554 |
| 25 hydroxyvitamin D | rs78649910 | 1.57E-09 | T | A | 0.0193309 | 0.00320205 | 0.893826 |
| 25 hydroxyvitamin D | rs8018720 | 1.95E-50 | G | C | 0.0383637 | 0.0025687 | 0.176691 |
| 25 hydroxyvitamin D | rs804281 | 2.48E-15 | A | G | -0.0157516 | 0.00199025 | 0.416402 |
| 25 hydroxyvitamin D | rs8063565 | 2.68E-08 | G | C | -0.0123302 | 0.00221722 | 0.266401 |
| 25 hydroxyvitamin D | rs8107974 | 1.49E-25 | A | T | -0.0386164 | 0.00369585 | 0.923752 |
| 25 hydroxyvitamin D | rs8121940 | 1.69E-52 | C | G | 0.037716 | 0.00247344 | 0.804752 |
| 25 hydroxyvitamin D | rs8181687 | 4.00E-09 | G | A | -0.0116958 | 0.00198761 | 0.42096 |
| 25 hydroxyvitamin D | rs9467550 | 3.36E-09 | A | G | 0.0188955 | 0.00319549 | 0.894762 |
| 25 hydroxyvitamin D | rs9476310 | 2.67E-09 | C | T | -0.0117234 | 0.00197015 | 0.488661 |
| 25 hydroxyvitamin D | rs964184 | 3.37E-50 | G | C | -0.0431884 | 0.00289883 | 0.131625 |
